# Risk of autism spectrum disorder and association of its symptoms with psychiatric and substance use disorders in non-clinical student sample in Kenya: cross-sectional study

**DOI:** 10.1192/bjo.2023.503

**Published:** 2023-08-22

**Authors:** Victoria N. Mutiso, David M. Ndetei, Esther N. Muia, Monicah Masake, Rita K. Alietsi, Lydia Onsinyo, Christine Musyimi, Daniel Mamah

**Affiliations:** Africa Mental Health Research and Training Foundation, Nairobi, Kenya; Africa Mental Health Research and Training Foundation, Nairobi, Kenya; Department of Psychiatry, University of Nairobi, Nairobi, Kenya; and World Psychiatric Association Collaborating Centre for Research and Training, Nairobi, Kenya; Department of Public and Community Health, Machakos University, Machakos, Kenya; Department of Psychiatry, Washington University Medical School, St Louis, Missouri, USA

**Keywords:** ASD, risk, patterns, psychiatric and substance use disorders, Kenyan students

## Abstract

**Background:**

The prevalence and patterns of autism spectrum disorder (ASD) symptoms/traits and the associations of ASD with psychiatric and substance use disorders has not been documented in non-clinical students in Sub-Saharan Africa, and Kenya in particular.

**Aims:**

To document the risk level of ASD and its traits in a Kenyan student population (high school, college and university) using the Autism-Spectrum Quotient (AQ); and to determine the associations between ASD and other psychiatric and substance use disorders.

**Method:**

This was a cross-sectional study among students (*n* = 9626). We used instruments with sufficient psychometric properties and good discriminative validity to collect data. A cut-off score of ≥32 on the AQ was used to identify those at high risk of ASD. We conducted the following statistical tests: (a) basic descriptive statistics; (b) chi-squared tests and Fisher's exact tests to analyse associations between categorical variables and ASD; (c) independent *t*-tests to examine two-group comparisons with ASD; (d) one-way analysis of variance to make comparisons between categorical variables with three or more groups and ASD; (e) statistically significant (*P* < 0.05) variables fitted into an ordinal logistic regression model to identify determinants of ASD; (f) Pearson's correlation and reliability analysis.

**Results:**

Of the total sample, 54 (0.56%) were at high risk of ASD. Sociodemographic differences were found in the mean scores for the various traits, and statistically significant (*P* < 0.05) associations we found between ASD and various psychiatric and substance use disorders.

**Conclusions:**

Risk of ASD, gender characteristics and associations with psychiatric and substance use disorders are similar in this Kenyan sample to those found in Western settings in non-clinical populations.

Autism spectrum disorder (ASD) is a developmental disorder of growing global public health concern that presents with some of the greatest burdens of disease in children and adolescents.^1^ The externalising behaviours of ASD may contribute to its greater manifestation in boys than in girls, thus accounting for the observed higher prevalence of ASD in boys than in girls.^2^ ASD occurs among African children living both in Africa and abroad.^3–7^ Most studies in Sub-Saharan Africa have come from paediatric neurology clinics, where the most common comorbid disorders were neurological and developmental conditions or intellectual disability; for example, the prevalence of ASD in a paediatric neurology clinic in Nigeria was 2.3%^8^ and in Nigerian children with intellectual disability it was 11.4%.^9^ To the best of our knowledge, the only community survey of ASD in Sub-Saharan Africa, involving 1169 children aged between 2 and 9 years^10^ in Uganda, found a prevalence of 0.68%, which is comparable to global estimates^11^ but lower than the 1.85% reported in the USA.^12^ In particular, we have not found any study in non-clinical high school, college and university students reported in Sub-Saharan Africa. ASD is associated with a wide variety of psychiatric disorders, including psychosis and, in particular, schizophrenia,^13,14^ bipolar disorder,^15^ developmental disorders in childhood and psychotic experiences in adolescence^16^ and post-traumatic stress disorder (PTSD) following exposure to traumatic life events and social victimisation.^17^ Traumatic events and social victimisation can lead to various psychiatric disorders, including depression, anxiety, bipolar disorders, personality disorders, psychotic disorders and trauma-related disorders, especially post-traumatic stress disorder.^18^ Individuals with ASD are more likely to show obsessive–compulsive traits, and people with obsessive–compulsive disorder (OCD) are more likely to show autistic symptoms.^19,20^ There is evidence of increased rates of substance use disorders among individuals with ASD.^21^ We have not found any community-based studies in non-clinical populations that report the comorbidity of ASD and psychiatric and substance use disorders.

The aims of the study were to: (a) document the overall ASD risk level and its traits/symptoms in a Kenyan student population and (b) determine the associations between ASD, psychiatric disorders and substance use disorders.

## Method

### Recruitment and data collection

This was a cross-sectional study. Participants were recruited from 4 out of the 47 counties in Kenya: Nairobi (the capital city of Kenya) and Machakos, Kitui and Makueni (three counties in the south-east of the country). Participants were high school, college and university students. High school participants were recruited from a community in Machakos County. We were already working with these four counties on other projects and had gained acceptance from local people, hence the choice of these counties for this project. College and university students who were ≥18 years and could communicate in English were invited to participate in the study after the nature of the study had been explained to them following one of their lectures in a classroom setting. The choice of the high school was leveraged on another study that had already taken place in the same communities. Since the schools were closed, we went through the community administrative structures to reach the families. We explained the nature of the study to the parents and guardians and, for those willing for their children (aged <18 years) to participate, we obtained written informed consent. The children were then invited to a central place where the nature of the study was explained and assent obtained from them. Only those children who presented themselves at the community meeting places were invited to participate in the study. The focus of the study was on students who are equitably admitted through a central process regardless of which county they come from and regardless of the socioeconomic and urban/rural status of the different counties in which the educational establishments were located. The only exclusion criteria for those approached in a classroom setting or who had come to the community centre for data collection were (a) no consent for those aged ≥18 years, (b) no parental consent for those <18 years and (c) no assent by those <18 years. A total of 9626 participants were recruited for the study.

### Ethics statement

The authors assert that all procedures contributing to this work comply with the ethical standards of the relevant national and institutional committees on human experimentation and with the Helsinki Declaration of 1975, as revised in 2008. Ethical approval was granted by the Maseno University Ethics Review Board in Kenya (IRB number MSU/DRPI/MUERC/00344/16).

### Instruments

#### Sociodemographic characteristics

A researcher designed a sociodemographic questionnaire to capture the sociodemographic data.

#### Autism-Spectrum Quotient (AQ)

This is a self-assessment questionnaire that evaluates the following autistic traits: social skills, communication skills, imagination, attention to detail and attention switching/tolerance to change.^22^ Total scores range between 0 and 50. A score ≥32 indicates a strong likelihood of an autism spectrum disorder,^22,23^ whereas a score of 26–31 indicates low risk. Thus, the AQ will identify individual symptoms of autism spectrum, and computation for the various symptoms can give a diagnosis of autism spectrum disorder.

#### Psychiatric Diagnostic Screening Questionnaire (PDSQ**)**

This is a self-report questionnaire that has 126 questions assessing symptoms of 13 DSM-IV Axis I disorders: major depression; anxiety disorders (panic disorder, agoraphobia, post-traumatic stress disorder (PTSD), obsessive–compulsive disorder, generalised anxiety disorder and social phobia); substance use disorders (alcohol misuse/dependence and drug misuse/dependence); and somatoform disorders (somatisation disorder and hypochondriasis). It has an additional six-item psychosis screen. Six questions on major depressive episode measure suicidal ideation, classified as: frequently thinking of dying in passive ways like going to sleep and not waking up, wishing to be dead, thinking you were better off dead, having thoughts of suicide, seriously considering taking life, and thinking about a specific way of taking your life. The questions are coded No/Yes, with values of zero and one respectively. The PDSQ has sufficient psychometric properties and almost excellent internal consistency. In a validity study that involved 994 psychiatric out-patients,^24^ Cronbach's α was greater than 0.80 for 12 of the 13 subscales, and the mean of the α coefficient was 0.86. Good to excellent levels of internal consistency, test–retest reliability and discriminant and convergent validity were achieved in previous studies.^27^ However, its psychometric properties have not been tested in a sociocultural context similar to Kenya. In mitigation, the PDSQ screens for symptoms that are diagnostic of DSM-IV disorders.

#### Washington Early Recognition Center Affectivity and Psychosis (WERCAP) screen

The WERCAP is a tool for assessing risk of developing a bipolar or psychotic disorder, based on severity of mood symptoms (‘affectivity’) and psychotic symptoms, using both frequency of occurrence and effect on functioning. It has high test–retest reliability and validity, with sensitivity of 0.91 and specificity of 0.71 for affectivity and sensitivity of 0.88 and specificity of 0.82 for psychosis.^25^ Its sufficient psychometric properties have been validated in Rwanda and Kenya.^26,27^

#### Alcohol, Smoking and Substance Involvement Screening Test (ASSIST)

The ASSIST collects information on the use of tobacco products, alcohol, cannabis, amphetamine-type stimulants, cocaine, sedatives and sleeping pills, hallucinogens, opioids and ‘other’ drugs, and determines levels of risk from the use of these substances.^28^ This instrument was developed by the World Health Organization specifically for use in low-resource settings such as Kenya. It has sufficient psychometric properties and good discriminative validity. It can adequately screen for low-, moderate- and high-risk use of any substance. From a test–retest reliability study conducted in nine different countries that involved 1047 respondents, Cronbach's α was over 0.80.^29^

### Training on data collection

Training on data collection was given by D.M. (from Washington University, St. Louis) in face-to-face residential training in Kenya. Data collection was overseen by D.M.N. and V.M. (from the Africa Mental Health Research and Training Foundation, AMHRTF). The lead research assistant, Isaiah Gitonga, was a university Masters Level graduate nurse who was responsible for the supervision of all sites and all data collection and transmission to AMHRTF headquarters. He supervised informed consent and sat through all data collection sessions.

### Data management and analysis

The coded data were checked, cleaned and exported into SPSS version 23 for Windows for analysis. Basic descriptive statistics (frequency, percentages, means and standard deviations) were calculated. The chi-squared test and Fisher's exact test when appropriate were used for categorical data to analyse the association of the various variables with the ASD risk level. For continuous data, the independent *t*-test and one-way analysis of variance were used to examine the association of sociodemographic variables with AQ scores. Significant PDSQ disorders, risk levels of substance use and sociodemographic variables were then fitted to a generalised linear model (GLM) with multinomial distribution and cumulative negative log–log link function to identify the predictors of ASD in the participants. Correlation analysis was also carried out between AQ total score and PDSQ disorder scores, AQ total score and substance use scores, and AQ total score and AQ subscale scores. The reliability of the various AQ subscales was indexed using Cronbach's alphas (internal consistency). Statistical significance was considered at *P* < 0.05.

## Results

A total of the 9626 students were approached and all participated in the study.

### Reliability of the AQ

Internal consistency coefficients and cross-subscale correlations are displayed in Table 1.

**Table 1:** Descriptive statistics for the Autism-Spectrum Quotient (AQ) test in a student sample (*n* = 9626)

AQ	Total AQ score, mean (s.d.)	Reliability, Cronbach's α	Pearson correlations					
			AQ total score	Social skills	Attention switching	Attention to detail	Communication	Imagination
AQ total score	20.78 (4.31)	0.91	1					
Social skills	3.47 (1.78)	0.65	0.583*	1				
Attention switching	4.97 (1.46)	0.68	0.440*	0.093*	1			
Attention to detail	4.79 (1.80)	0.72	0.373*	−0.122*	−0.017	1		
Communication	3.71 (1.78)	0.72	0.655*	0.243*	0.135*	0.076*	1	
Imagination	3.84 (1.52)	0.66	0.522*	0.251*	0.041*	−0.052*	0.179*	1

* Correlation is significant at the 0.01 level (2-tailed).

The internal consistency coefficients (Cronbach's alphas) were generally satisfactory (mean α = 0.72). Cronbach's alpha for AQ total score was notably high. Cronbach's alpha for social skills, attention switching and imagination were notably low.

Correlations among AQ subscales were weak to moderate (ρ = 0.041–0.251). Note also that attention to detail correlated inversely with social skills and imagination.

### ASD risk level

Of the 9626 participants, 54 (0.56%) were found to be at high risk of ASD and 1255 (13.0%) at low risk.

### Sociodemographic characteristics and associated AQ scores

#### Distribution of sociodemographic variables

Males (53.5%) were predominant among the 9626 respondents. The mean age was 21.45 years (s.d. = 2.30, median 21.36). The majority of respondents were single (93.3%), attending university (69.4%), were either first or second born in their families (56.9%) and were Protestant (57.1%).

#### Sociodemographic factors associated with the risk of ASD

Sociodemographic factors found to be associated with the risk of ASD are summarised in Table 2. Males were at a significantly higher risk of ASD compared with females (*P* < 0.001). Of the religions, only Protestants were significantly associated with high risk of ASD (*P* = 0.021). Being first or second born and a high school student was also significantly (*P* < 0.05) associated with a high risk of ASD, as was an age of 19 or younger (including, of course, high school students). Of the demographics, only marital status was not significantly associated with high ASD risk.

**Table 2:** Sociodemographic factors associated with risk of autism spectrum disorder (ASD) in a student sample (*n* = 9626)

Variable	Category	Total	ASD risk*			*P*	Cramer's *V*
			No risk (AQ < 26)	Low risk (AQ = 26–31)	High risk (AQ ≥ 32)		
Gender	Male	5111 (53.5%)	4341 (52.6%)^a^	731 (58.5%)^b^	39 (72.2%)^c^	**<0.001**^d^	0.05
	Female	4446 (46.5%)	3912 (47.4%)^a^	519 (41.5%)^b^	15 (27.8%)^c^		
Age, years	≤19	2335 (24.3%)	1940 (23.3%)^a^	381 (30.4%)^b^	14 (25.9%)^a,b^	**<0.001**^d^	0.04
	20–24	6807 (70.7%)	5961 (71.7%)^a^	811 (64.6%)^b^	35 (64.8%)^a,b^		
	≥25	484 (5.03%)	416 (5.00%)^a^	63 (5.02%)^a^	5 (9.26%)^a^		
Marital status	Married	601 (6.27%)	508 (6.13%)^a^	90 (7.23%)^a^	3 (5.56%)^a^	0.294^e^	0.02
	Single	8948 (93.3%)	7750 (93.5%)^a^	1147 (92.1%)^a^	51 (94.4%)^a^		
	Others	38 (0.40%)	30 (0.36%)^a^	8 (0.64%)^a^	0 (0%)^a^		
Religion	Protestant	5444 (57.1%)	4743 (57.6%)^a^	669 (53.5%)^b^	32 (59.3%)^a,b^	**0.021**^e^	0.03
	Catholic	3314 (34.8%)	2828 (34.4%)^a^	470 (37.6%)^b^	16 (29.6%)^a,b^		
	Muslim	409 (4.29%)	345 (4.19%)^a^	58 (4.64%)^a^	6 (11.1%)^b^		
	Other	367 (3.85%)	314 (3.82%)^a^	53 (4.24%)^a^	0 (0%)^a^		
Birth order	1–2	5467 (56.9%)	4665 (56.2%)^a^	771 (61.5%)^b^	31 (57.4%)^a,b^	**0.011**^d^	0.03
	3–5	3232 (33.6%)	2834 (34.1%)^a^	380 (30.3%)^b^	18 (33.3%)^a,b^		
	≥6	915 (9.52%)	808 (9.73%)^a^	102 (8.14%)^a^	5 (9.26%)^a^		
Education level	High school	1394 (14.6%)	1078 (13.0%)^a^	307 (24.7%)^b^	9 (16.7%)^a,b^	**<0.001**^d^	0.08
	College	1534 (16.0%)	1335 (16.1%)^a^	188 (15.1%)^a^	11 (20.4%)^a^		
	University	6644 (69.4%)	5864 (70.8%)^a^	746 (60.1%)^b^	34 (63.0%)^a,b^		

AQ, Autism-Spectrum Quotient; bold denotes significance at *P* < 0.05.

a.–c. Each superscript letter (a–c) denotes a subset of ASD risk categories whose column proportions do not differ significantly from each other at the 0.05 level.

d. chi-squared test

e. Fisher's exact test.

#### Sociodemographic differences in AQ scores

Sociodemographic differences in the different traits of ASD are summarised in Table 3.

**Table 3:** Sociodemographic differences in the five Autism-Spectrum Quotient subscales in a student sample (*n* = 9626)

Variable	Category	Autism Spectrum Quotient																	
		Total score			Social skills			Attention switching			Attention to detail			Communication			Imagination		
		Mean (s.d.)	*P*	Effect size	Mean (s.d.)	*P*	Effect size	Mean (s.d.)	*P*	Effect size	Mean (s.d.)	*P*	Effect size	Mean (s.d.)	*P*	Effect size	Mean (s.d.)	*P*	Effect size
Gender^a^	Male	21.00 (4.32)	**<0.001**	0.110	3.40 (1.78)	**<0.001**	−0.090	4.97 (1.46)	0.647	−0.010	4.91 (1.79)	**<0.001**	0.130	3.83 (1.79)	**<0.001**	0.140	3.90 (1.51)	**<0.001**	0.090
	Female	20.54 (4.30)			3.55 (1.77)			4.98 (1.46)			4.67 (1.80)			3.57 (1.77)			3.77 (1.53)		
Age, years^b^	≤19	21.28 (4.31)	**<0.001**	0.004	3.69 (1.84)	**<0.001**	0.005	5.00 (1.46)	**0.028**	0.001	4.81 (1.74)	**0.042**	0.001	3.91 (1.79)	**<0.001**	0.004	3.87 (1.55)	0.230	0.0003
	20–24	20.61 (4.30)			3.40 (1.75)			4.98 (1.46)			4.78 (1.82)			3.64 (1.78)			3.82 (1.51)		
	≥25	20.82 (4.25)			3.44 (1.85)			4.81 (1.44)			4.99 (1.70)			3.68 (1.83)			3.91 (1.48)		
Marital status^b^	Married	21.22 (4.33)	**<0.001**	0.002	3.61 (1.69)	**0.022**	0.001	4.94 (1.39)	0.648	0.0001	4.82 (1.75)	0.579	0.0001	3.91 (1.86)	**0.007**	0.001	3.95 (1.44)	**0.016**	0.001
	Single	20.74 (4.30)			3.46 (1.78)			4.97 (1.46)			4.79 (1.80)			3.69 (1.78)			3.83 (1.52)		
	Others	22.71 (4.07)			4.03 (2.05)			5.16 (1.31)			5.08 (1.50)			4.08 (1.75)			4.37 (1.62)		
Religion^b^	Protestant	20.68 (4.29)	**0.027**	0.001	3.45 (1.79)	0.314	0.0004	4.95 (1.47)	0.086	0.001	4.78 (1.82)	0.064	0.001	3.65 (1.78)	**0.001**	0.002	3.83 (1.53)	0.345	0.0003
	Catholic	20.94 (4.31)			3.49 (1.77)			4.98 (1.43)			4.82 (1.75)			3.79 (1.78)			3.87 (1.52)		
	Muslim	20.75 (4.63)			3.58 (1.82)			5.07 (1.50)			4.61 (1.79)			3.62 (1.82)			3.87 (1.51)		
	Other	21.02 (4.34)			3.38 (1.79)			5.12 (1.47)			4.93 (1.91)			3.87 (1.76)			3.72 (1.39)		
Birth order^b^	1–2	20.90 (4.36)	**0.011**	0.001	3.51 (1.81)	**0.027**	0.001	4.99 (1.49)	0.631	0.0001	4.82 (1.81)	0.073	0.001	3.73 (1.78)	0.149	0.0004	3.84 (1.52)	0.838	0.00004
	3–5	20.65 (4.27)			3.42 (1.75)			4.96 (1.41)			4.78 (1.77)			3.66 (1.80)			3.84 (1.51)		
	≥6	20.57 (4.11)			3.39 (1.70)			4.97 (1.46)			4.68 (1.80)			3.71 (1.77)			3.81 (1.49)		
Education level^b^	High school	22.35 (4.09)	**<0.001**	0.023	3.93 (1.79)	**<0.001**	0.012	5.04 (1.42)	**0.005**	0.001	4.91 (1.66)	**0.023**	0.001	4.25 (1.72)	**<0.001**	0.016	4.21 (1.51)	**<0.001**	0.011
	College	20.72 (4.31)			3.36 (1.77)			5.05 (1.44)			4.82 (1.77)			3.68 (1.78)			3.81 (1.52)		
	University	20.46 (4.28)			3.39 (1.76)			4.94 (1.47)			4.77 (1.83)			3.60 (1.78)			3.77 (1.51)		

Bold denotes significance at *P* < 0.05.

a. Independent *t*-test/Cohen's *D* statistic.

b. One-way analysis of variance/eta-squared statistic.

Males had significantly higher AQ mean scores compared with females. Social skills scores were significantly higher in females than in males. Attention to detail, communication and imagination scores were significantly higher in males than females. There was no significant difference in attention switching scores. Overall, males had more ASD traits.

Younger age was significantly associated with higher AQ mean scores compared with older age. Social skills, attention switching and communication scores were significantly higher in younger participants. Attention to detail mean score was significantly higher in older participants. There was no significant between-group difference in imagination. Overall, those in the younger age group had more ASD traits.

High school students had significantly higher AQ mean scores compared with college and university students. Social skills, attention to detail, communication and imagination scores were significantly higher in high school students than in college and university students. Attention switching was significantly lower in university students than in high school and college students. Overall, high school students had more ASD traits.

### Psychiatric disorders (PDSQ and WERCAP) associated with ASD risk

Table 4 summarises the association between risk of ASD and psychiatric disorders on the PDSQ and WERCAP. Significant differences (*P* < 0.05) were observed between those at risk of ASD and all the listed psychiatric disorders. Progressively, high risk of psychosis increased from 4.02% for no risk of ASD through 7.81% for low risk of ASD to 16.7% for high risk of ASD, which is much higher than the average risk of 4.58%. Other psychiatric disorders follow the same pattern of progressively increasing risk of ASD. OCD stands out as the disorder with the highest progressively increasing risk of ASD.

**Table 4:** Psychiatric disorders (PDSQ and WERCAP) associated with risk of autism spectrum disorder (ASD) in a student sample (*n* = 9626)

Disorder	Presence or risk status	Total	ASD risk			χ^2^ test, *P*	Cramer's *V*
			No risk (AQ < 26)	Low risk (AQ = 26–31)	High risk (AQ ≥ 32)		
**PDSQ**							
Major depressive disorder	No	7608 (79.0%)	6712 (80.7%)^a^	868 (69.2%)^b^	28 (51.9%)^c^	**<0.001**	0.11
	Yes	2018 (21.0%)	1605 (19.3%)^a^	387 (30.8%)^b^	26 (48.1%)^c^		
Post-traumatic stress disorder	No	7008 (72.8%)	6166 (74.1%)^a^	810 (64.5%)^b^	32 (59.3%)^b^	**<0.001**	0.08
	Yes	2618 (27.2%)	2151 (25.9%)^a^	445 (35.5%)^b^	22 (40.7%)^b^		
Bulimia/binge eating disorder	No	9312 (96.7%)	8084 (97.2%)^a^	1177 (93.8%)^b^	51 (94.4%)^a,b^	**<0.001**	0.07
	Yes	313 (3.25%)	232 (2.79%)^a^	78 (6.22%)^b^	3 (5.56%)^a,b^		
Obsessive–compulsive disorder	No	3385 (35.2%)	2964 (35.6%)^a^	412 (32.8%)^a^	9 (16.7%)^b^	**0.003**	0.04
	Yes	6240 (64.8%)	5352 (64.4%)^a^	843 (67.2%)^a^	45 (83.3%)^b^		
Panic disorder	No	7753 (80.6%)	6811 (81.9%)^a^	908 (72.4%)^b^	34 (63.0%)^b^	**<0.001**	0.09
	Yes	1871 (19.4%)	1504 (18.1%)^a^	347 (27.6%)^b^	20 (37.0%)^b^		
Psychosis	No	5639 (58.6%)	5008 (60.2%)^a^	610 (48.6%)^b^	21 (38.9%)^b^	**<0.001**	0.09
	Yes	3986 (41.4%)	3308 (39.8%)^a^	645 (51.4%)^b^	33 (61.1%)^b^		
Agoraphobia	No	6319 (65.7%)	5563 (66.9%)^a^	727 (57.9%)^b^	29 (53.7%)^b^	**<0.001**	0.07
	Yes	3306 (34.3%)	2753 (33.1%)^a^	528 (42.1%)^b^	25 (46.3%)^b^		
Social phobia	No	4791 (49.8%)	4247 (51.1%)^a^	527 (42.0%)^b^	17 (31.5%)^b^	**<0.001**	0.07
	Yes	4835 (50.2%)	4070 (48.9%)^a^	728 (58.0%)^b^	37 (68.5%)^b^		
Alcohol misuse/dependence	No	7461 (77.5%)	6587 (79.2%)^a^	838 (66.8%)^b^	36 (66.7%)^b^	**<0.001**	0.10
	Yes	2165 (22.5%)	1730 (20.8%)^a^	417 (33.2%)^b^	18 (33.3%)^b^		
Drug misuse/dependence	No	7983 (82.9%)	7044 (84.7%)^a^	901 (71.8%)^b^	38 (70.4%)^b^	**<0.001**	0.12
	Yes	1643 (17.1%)	1273 (15.3%)^a^	354 (28.2%)^b^	16 (29.6%)^b^		
Generalised anxiety disorder	No	8335 (86.6%)	7254 (87.2%)^a^	1041 (82.9%)^b^	40 (74.1%)^b^	**<0.001**	0.05
	Yes	1291 (13.4%)	1063 (12.8%)^a^	214 (17.1%)^b^	14 (25.9%)^b^		
Somatisation disorder	No	7151 (74.3%)	6269 (75.4%)^a^	847 (67.5%)^b^	35 (64.8%)^a,b^	**<0.001**	0.06
	Yes	2475 (25.7%)	2048 (24.6%)^a^	408 (32.5%)^b^	19 (35.2%)^a,b^		
Hypochondriasis	No	7016 (72.9%)	6213 (74.7%)^a^	775 (61.8%)^b^	28 (51.9%)^b^	**<0.001**	0.10
	Yes	2610 (27.1%)	2104 (25.3%)^a^	480 (38.2%)^b^	26 (48.1%)^b^		
**WERCAP**							
Psychosis	Low risk	9185 (95.4%)	7983 (96.0%)^a^	1157 (92.2%)^b^	45 (83.3%)^c^	**<0.001**	0.08
	High risk	441 (4.58%)	334 (4.02%)^a^	98 (7.81%)^b^	9 (16.7%)^c^		

Bold denotes significance at *P* < 0.05.

PDSQ, Psychiatric Diagnostic Screening Questionnaire; WERCAP, Washington Early Recognition Center Affectivity and Psychosis screen.

a.–c. Each superscript letter (a–c) denotes a subset of ASD risk categories whose column proportions do not differ significantly from each other at the 0.05 level.

There were significantly positive correlations between AQ total score and all psychiatric symptom scores on the PDSQ (*P* < 0.01). The correlation ranged from *r* = 0.111 to *r* = 0.207; the highest correlation was with the psychosis score, followed by major depressive disorder and eating disorder, and the lowest correlation was with OCD. More detailed data are presented in Table 5.

**Table 5:** Correlation between Autism-Spectrum Quotient (AQ) total score and Psychiatric Diagnostic Screening Questionnaire (PDSQ) scores

Pearson's correlation	1	2	3	4	5	6	7	8	9	10	11	12	13	14	15
1 AQ total score	1														
2 Major depressive disorder score	0.194*	1													
3 PTSD score	0.144*	0.513*	1												
4 Bulimia/binge eating disorder score	0.193*	0.487*	0.410*	1											
5 Obsessive–compulsive disorder score	0.111*	0.425*	0.388*	0.376*	1										
6 Panic disorder score	0.184*	0.497*	0.448*	0.441*	0.524*	1									
7 Psychosis score	0.207*	0.465*	0.441*	0.464*	0.473*	0.560*	1								
8 Agoraphobia score	0.156*	0.431*	0.388*	0.394*	0.460*	0.501*	0.470*	1							
9 Social phobia score	0.134*	0.460*	0.374*	0.362*	0.467*	0.451*	0.422*	0.572*	1						
10 Alcohol misuse/ dependence score	0.149*	0.314*	0.266*	0.377*	0.209*	0.296*	0.332*	0.283*	0.273*	1					
11 Drug misuse/ dependence score	0.151*	0.294*	0.264*	0.370*	0.197*	0.257*	0.311*	0.246*	0.231*	0.616*	1				
12 Generalised anxiety disorder score	0.129*	0.518*	0.411*	0.374*	0.423*	0.485*	0.422*	0.467*	0.554*	0.273*	0.273*	1			
13 Somatisation disorder score	0.138*	0.409*	0.331*	0.344*	0.300*	0.391*	0.353*	0.376*	0.374*	0.296*	0.288*	0.422*	1		
14 Hypochondriasis score	0.157*	0.394*	0.335*	0.347*	0.300*	0.421*	0.379*	0.375*	0.363*	0.302*	0.312*	0.449*	0.525*	1	
15 Psychosis (WERCAP) score	0.190*	0.486*	0.381*	0.350*	0.351*	0.393*	0.444*	0.338*	0.325*	0.241*	0.231*	0.383*	0.290*	0.312*	1

PTSD, post-traumatic stress disorder; WERCAP, Washington Early Recognition Center Affectivity and Psychosis screen.

* Correlation is significant at the 0.01 level (2-tailed).

### Association of ASD risk with ASSIST diagnoses of substance use

As reported in Table 6, all drugs/substances were significantly (*P* < 0.05) associated with ASD risk except for alcohol and cannabis.

**Table 6:** Risk levels of ASSIST individual substance uses associated with risk of autism spectrum disorder (ASD) in a student sample (*n* = 9626)

Drug	Risk level of substance use	Total	ASD risk*			Fisher's exact test, *P*	Cramer's V
			No risk (AQ < 26)	Low risk (AQ = 26–31)	High risk (AQ ≥ 32)		
Alcohol	Low risk	1502 (59.3%)	1336 (59.7%)^a^	156 (56.5%)^a^	10 (55.6%)^a^	0.504	0.02
	Moderate risk	817 (32.3%)	721 (32.2%)^a^	90 (32.6%)^a^	6 (33.3%)^a^		
	High risk	214 (8.45%)	182 (8.13%)^a^	30 (10.9%)^a^	2 (11.1%)^a^		
Tobacco	Low risk	1941 (77.9%)	1734 (78.7%)^a^	191 (70.2%)^b^	16 (94.1%)^a^	**0.008**	0.05
	Moderate risk	520 (20.9%)	444 (20.2%)^a^	75 (27.6%)^b^	1 (5.88%)^a^		
	High risk	30 (1.20%)	24 (1.09%)^a^	6 (2.21%)^a^	0 (0%)^a^		
Cannabis	Low risk	1983 (80.2%)	1765 (80.7%)^a^	205 (75.9%)^a^	13 (76.5%)^a^	0.089	0.04
	Moderate risk	429 (17.3%)	373 (17.1%)^a^	52 (19.3%)^a^	4 (23.5%)^a^		
	High risk	62 (2.51%)	49 (2.24%)^a^	13 (4.81%)^b^	0 (0%)^a,b^		
Cocaine	Low risk	2329 (94.9%)	2074 (95.6%)^a^	239 (89.2%)^b^	16 (94.1%)^a,b^	**<0.001**	0.09
	Moderate risk	113 (4.60%)	90 (4.15%)^a^	22 (8.21%)^b^	1 (5.88%)^a,b^		
	High risk	12 (0.49%)	5 (0.23%)^a^	7 (2.61%)^b^	0 (0%)^a,b^		
Amphetamines	Low risk	2328 (94.9%)	2074 (95.7%)^a^	238 (88.8%)^b^	16 (94.1%)^a,b^	**<0.001**	0.08
	Moderate risk	113 (4.61%)	87 (4.01%)^a^	25 (9.33%)^b^	1 (5.88%)^a,b^		
	High risk	12 (0.49%)	7 (0.32%)^a^	5 (1.87%)^b^	0 (0%)^a,b^		
Inhalants	Low risk	2349 (95.6%)	2093 (96.4%)^a^	239 (89.2%)^b^	17 (100.0%)^a,b^	**<0.001**	0.09
	Moderate risk	98 (3.99%)	74 (3.41%)^a^	24 (8.96%)^b^	0 (0%)^a,b^		
	High risk	9 (0.37%)	4 (0.18%)^a^	5 (1.87%)^b^	0 (0%)^a,b^		
Sedatives	Low risk	2274 (92.0%)	2030 (92.8%)^a^	229 (85.1%)^b^	15 (88.2%)^a,b^	**<0.001**	0.08
	Moderate risk	180 (7.28%)	146 (6.68%)^a^	32 (11.9%)^b^	2 (11.8%)^a,b^		
	High risk	19 (0.77%)	11 (0.50%)^a^	8 (2.97%)^b^	0 (0%)^a,b^		
Hallucinogens	Low risk	2349 (95.6%)	2089 (96.3%)^a^	244 (90.7%)^b^	16 (88.9%)^a,b^	**<0.001**	0.08
	Moderate risk	94 (3.83%)	74 (3.41%)^a^	18 (6.69%)^b^	2 (11.1%)^a,b^		
	High risk	13 (0.53%)	6 (0.28%)^a^	7 (2.60%)^b^	0 (0%)^a,b^		
Opioids	Low risk	2354 (95.9%)	2099 (96.8%)^a^	239 (88.8%)^b^	16 (94.1%)^a,b^	**<0.001**	0.10
	Moderate risk	90 (3.67%)	65 (3.00%)^a^	24 (8.92%)^b^	1 (5.88%)^a,b^		
	High risk	11 (0.45%)	5 (0.23%)^a^	6 (2.23%)^b^	0 (0%)^a,b^		

ASSIST, Alcohol, Smoking, and Substance Involvement Screening Test; bold denotes significance at *P* < 0.05.

a., b. each superscript (a, b) letter denotes a subset of ASD risk categories whose column proportions do not differ significantly from each other at the 0.05 level.

There were significant positive correlations between AQ total score and all drug/substance use scores (*P* < 0.01). More detailed data are presented in Table 7.

**Table 7:** Correlation between ASSIST and Autism-Spectrum Quotient (AQ) total score

Pearson's correlations	1	2	3	4	5	6	7	8	9	10
1 AQ total score	1									
2 Tobacco	0.109*	1								
3 Alcohol	0.064*	0.461*	1							
4 Cannabis	0.082*	0.457*	0.418*	1						
5 Cocaine	0.097*	0.194*	0.131*	0.329*	1					
6 Amphetamines	0.109*	0.140*	0.107*	0.237*	0.546*	1				
7 Inhalants	0.126*	0.217*	0.148*	0.281*	0.590*	0.603*	1			
8 Sedatives	0.114*	0.130*	0.142*	0.203*	0.424*	0.553*	0.510*	1		
9 Hallucinogens	0.125*	0.141*	0.129*	0.220*	0.555*	0.536*	0.653*	0.494*	1	
10 Opioids	0.115*	0.189*	0.154*	0.298*	0.543*	0.542*	0.613*	0.471*	0.687*	1

ASSIST, Alcohol, Smoking, and Substance Involvement Screening Test.

* Correlation is significant at the 0.01 level (2-tailed).

### Determinants of ASD risk

These are summarised in Table 8. Major depressive disorder and drug misuse/dependence are determinants of ASD risk (*P* < 0.05). The risk of ASD was 1.45 and 1.83 times higher in participants with major depressive disorder and drug misuse/dependence respectively.

**Table 8:** Factors associated with autism spectrum disorder: ordinal logistic regression model

Variable	Category	AOR	95% CI	*P*
Gender	Male	1.27	0.94–1.72	0.125
	Female	Ref.		
Age group, years	≤19	1.14	0.64–2.14	0.664
	20–24	0.76	0.44–1.36	0.325
	≥25	Ref.		
Religion	Protestant	Ref.		
	Catholic	1.19	0.90–1.57	0.209
	Muslim	1.60	0.77–3.08	0.181
	Other	1.32	0.67–2.44	0.389
Birth position	1–2	1.35	0.83–2.28	0.244
	3–5	0.93	0.55–1.62	0.788
	≥6	Ref.		
Level of education	High School	1.18	0.79–1.74	0.400
	College	0.87	0.59–1.26	0.480
	University	Ref.		
Major depressive disorder	No	Ref.		
	Yes	1.45	1.04–2.02	**0.027**
Post-traumatic stress disorder	No	Ref.		
	Yes	1.18	0.86–1.61	0.310
Bulimia/binge eating disorder	No	Ref.		
	Yes	0.75	0.41–1.31	0.331
Obsessive–compulsive disorder	No	Ref.		
	Yes	0.74	0.52–1.05	0.086
Panic disorder	No	Ref.		
	Yes	1.21	0.86–1.71	0.275
Psychosis	No	Ref.		
	Yes	1.18	0.84–1.65	0.338
Agoraphobia	No	Ref.		
	Yes	0.90	0.64–1.24	0.513
Social phobia	No	Ref.		
	Yes	1.35	0.97–1.88	0.079
Alcohol misuse/dependence	No	Ref.		
	Yes	0.90	0.64–1.26	0.552
Drug misuse/dependence	No	Ref.		
	Yes	1.83	1.29–2.59	**<0.001**
Generalised anxiety disorder	No	Ref.		
	Yes	0.96	0.67–1.36	0.813
Somatisation disorder	No	Ref.		
	Yes	1.17	0.85–1.61	0.337
Hypochondriasis	No	Ref.		
	Yes	1.32	0.95–1.82	0.092
Psychosis (WERCAP)	Low risk	Ref.		
	High risk	1.46	0.91–2.28	0.107
Tobacco	Low risk	Ref.		
	Moderate risk	0.79	0.56–1.11	0.181
	High risk	0.84	0.25–2.33	0.757
Cocaine	Low risk	Ref.		
	Moderate risk	1.14	0.58–2.13	0.701
	High risk	2.57	0.51–12.13	0.234
Amphetamine	Low risk	Ref.		
	Moderate risk	1.15	0.58–2.19	0.676
	High risk	1.25	0.10–9.42	0.847
Inhalants	Low risk	Ref.		
	Moderate risk	1.10	0.51–2.23	0.808
	High risk	0.91	0.07–10.86	0.943
Sedatives	Low risk	Ref.		
	Moderate risk	1.17	0.66–1.99	0.568
	High risk	1.88	0.54–5.86	0.294
Hallucinogens	Low risk	Ref.		
	Moderate risk	0.85	0.38–1.78	0.669
	High risk	1.22	0.16–8.18	0.841
Opioids	Low risk	Ref.		
	Moderate risk	1.94	0.95–3.86	0.063
	High risk	1.61	0.21–10.52	0.626

AOR, adjusted odds ratio; Ref., reference variable; WERCAP, Washington Early Recognition Center Affectivity and Psychosis screen.

## Discussion

Our discussion is primarily based on what we found in our study. We were highly limited by the relative unavailability of data, particularly from Africa, for purposes of comparisons.

### Reliability of the AQ and its subscales

The fact that we found significant reliability of the AQ subscales implies that the results across the different students are reliable and provide a sound basis for comparison across all the variables studied.

### ASD risk level and sociodemographic variables

#### Global trends

As far as we could establish from our literature search, we present the largest sample (*n* = 9626) study reported in Africa on ASD risk in a population not drawn from clinical services; an earlier study, involving *n* = 1169, was reported in Kampala, Uganda,^10^ but with the difference that our study was on high school, college and university students whereas that in Uganda was on much younger children. This difference in age range between our study and the Ugandan study may be the explanation for our finding that 0.56% of participants were at high risk of ASD, compared with the higher 0.68% found in the Uganda children.^10^ Our finding of 0.56% is within the global range (0.3–1.16%, median 0.62%), but lower than the 1.85% reported in the USA.^12^ As expected, the 0.56% risk level is much lower than the 2.3% found in participants drawn from populations attending neurology clinics in Nigeria^8^ or 11.4% among participants with intellectual disability, also in Nigeria.^9^

#### Gender

The gender comparisons summarised in Tables 2 and 3 are similar to what has been found in most reported studies. For example, the finding that males had nearly three times the level of autistic traits compared with females (0.8% and 0.3% respectively) is similar to the 3:1 reported elsewhere.^30^ Females, however, had more impairment in social skills compared with males, although there were no gender differences in attention switching, a finding that has been observed in other studies.^30^

#### Age and level of education

The higher risk of ASD in the younger age group explains why in our study we found ASD to be significantly (*P* < 0.001) associated with high school students (who are younger than college and university students). It is not unexpected that high school students had higher levels of ASD risk, given that they are younger than college and university students. Students at high risk of ASD would naturally have dropped out because of the highly competitive academic entrance to colleges and universities. This explanation is further supported by Uganda's findings^10^ in younger children of a 0.68% prevalence of ASD, compared with the 0.56% average at high risk of ASD in our population, which included older students in higher learning institutions.

#### Marital status

It is noteworthy that marital status did not affect the risk of ASD. However, we do not have information on exactly what constituted ‘other' marital status. We speculate that this may have been a sensitive question that the students were not prepared to talk about. These could include students who are involved in ‘come we stay’ arrangements very common among college and university students for purposes of sharing the cost of accommodation. When it comes to specific symptoms of ASD, the students in the ‘other’ marital status group scored higher on poor social coping skills, poor communication and imagination. We have no explanation for this; only qualitative studies have the potential to provide the explanation.

#### Religion

We have no explanation for the marginally significant association of ASD risk with the Muslim religion. However, it is noteworthy that children of Somali background in Sweden, who are predominantly of Muslim background, had three times the risk of ASD compared with children of parents of Swedish background.^31^

#### Birth order

We speculate that the marginally significant association with first or second birth order, i.e. being the mother's first or second child, may be a reflection of brain injury associated with primigravida. Although we appreciate that pregnancy in older age could also be a risk factor for brain damage in the offspring, in this Kenya context we were dealing with a relatively young population of mothers and their first or second offspring.

### Association with psychiatric disorders

We found a highly significant association (*P* < 0.001) of ASD risk with all psychiatric disorders we studied except for OCD, for which the significance was *P* = 0.003, making OCD less associated than the other disorders. These statistically significant findings are confirmed by correlation analysis. It is to be noted that this association between psychiatric disorders and ASD is uniformly consistent for both low risk and high risk of ASD. Studies on biomarkers of ASD and psychiatric conditions, in particular schizophrenia, will clarify the relation between these conditions. It is also possible that school children and students with ASD are placed at a functional disadvantage that predisposes them to various psychiatric disorders, such as PTSD, as explained in our literature review.

### Association with substance misuse

Only alcohol and cannabis misuse did not achieve significant association with ASD risk. There was a consistent correlation between substance use and both low and high risk of ASD, observed all through and this is therefore likely to reflect the real trend, i.e. association between ASD and substance misuse. Further studies are required to confirm this.

### Determinants of ASD

In all cases of ASD, it is important to screen for depression and substance misuse as comorbidities, whether in clinical or non-clinical populations.

### Strengths and limitations of our study

The main strength of our study is that it is in the largest sample reported in Sub-Saharan Africa and is the first of its type in high school, college and university students. We used DSM-IV symptoms – DSM-IV is a widely used tool in both clinical and epidemiological research.

Our study had several limitations, although for some there were mitigating factors. The university students were drawn from 4 counties out of the 47 counties in Kenya. This study was leveraged on another study that was focused not on epidemiological patterns of psychiatric disorders but on biomarkers of early psychosis. It is therefore feasible that this approach may have introduced bias, as the sample may not be representative of the national sample. However, this is strongly mitigated by the admissions policy to public universities and colleges in Kenya. Placement in national universities is done centrally to ensure equitable distribution of students from all counties. We therefore believe that our sample of university students represented the face of Kenya.

The study in the high schools was leveraged on the same biomarker study. Unlike the universities, admission to high schools is partially regional, national and local. However, during this study, there was a nationwide closure of schools, with students going back to their homes and families. We were therefore forced to access only students who came from schools within the community, whom we approached collectively through the community administration to come to a central place. We have no reason to assume these schools were different from or similar to any other schools. Only future studies with a better sampling process will determine differences or similarities across a more representative sample. Either way, in the universities and schools, this study generates data that demonstrate that ASD is an ongoing concern in the Kenyan setting.

A major limitation of this study is that only individuals enrolled in typical educational settings participated. There is therefore the possibility that the study excluded individuals who were not enrolled in educational institutions.

Despite the strengths and limitations of our study, it remains essentially an exploratory descriptive study that at best serves to raise awareness of ASD in the non-clinical population, and in particular, students whose behavioural dysfunctions and possibly academic performance could be explained by the presence of unrecognised ASD.

## Data Availability

Data are available from the corresponding author on reasonable request.
